# Profile and outcomes of patients with acute complications of malaria presenting to an urban emergency department of a tertiary hospital in Tanzania

**DOI:** 10.1186/s13104-019-4388-8

**Published:** 2019-06-18

**Authors:** Raya Yusuph, Hendry R. Sawe, Paulina N. Nkondora, Juma A. Mfinanga

**Affiliations:** 1 Emergency Medicine Department, Muhimbili University of Health and Allied Sciences, P.O. Box 65001, Dar es Salaam, Tanzania; 2 Emergency Medicine Department, Muhimbili National Hospital, Dar es Salaam, Tanzania

**Keywords:** Malaria complications, Emergency department, Africa, Tanzania

## Abstract

**Objectives:**

In Tanzania, malaria ranks number three among the top ten causes of deaths in all age groups, however little is known about the utilisation of emergency department by patients with complications of malaria. We describe clinical presentation, resource utilization, and outcomes of acutely ill patients with complications of malaria presenting to an urban emergency department (ED) in Tanzania.

**Results:**

We screened 405 patients which physicians had a clinical suspicion or diagnosis of malaria at ED. We enrolled 184 (45.5%) patients meeting WHO clinical and laboratory definition of malaria. The median age was 22 years (interquartile range 22–33 years), 105 (57%) were male, and overall 124 (67.4%) were self-referral. The use of insecticide treated nets (ITNs) in this group was 125 (67.4%). Fever 125 (67.9%), headache 56 (30.4%) and general body malaise 41 (22.2%) were the top three frequent complains, while tachycardia 83 (42.9%) was the most frequent abnormal vital sign. Overall, 21 (11.4%) patients had severe anaemia and 21 (11.4%) had abnormal renal function test. In ED 121/184 (65.8%) patients received antimalarial, 74/184 (40.2%) received antibiotics, 6/184 (3.3%) received antipyretic/analgesic and 5/20 (25%) patients with severe anaemia received blood transfusion. Overall, 99/184 (53.8%) patients were hospitalized, 3 (1.6%) died at the ED, and the overall hospital morality was 3.8%. Overall we found a substantial burden of patients with complications of malaria presenting in the largest public ED in Tanzania.

**Electronic supplementary material:**

The online version of this article (10.1186/s13104-019-4388-8) contains supplementary material, which is available to authorized users.

## Introduction

Malaria is still a major health problem across the lower and middle income countries accounting for up to 40% of public expenditure in Africa [1]. In Africa about 300–500 million people suffer from malaria, of which millions of them progress to severe malaria. The Sub-Saharan Africa (SSA) is particularly most affected with malaria endemic, with estimates of 88% of global cases, mostly affecting children below the age of 5-years and pregnant women [2].

In Tanzania, as in other SSA countries, malaria is among the top health problems, affecting particularly the children below age of 5-years and pregnant women. Over 90% of the population in Tanzania lives in Malaria zones, and 60% of the country population lives in the malaria endemic zones [3, 4]. In Tanzania, malaria ranks number three among the top 10 causes of deaths in all age groups, but with as significant effect in children under the age of five, accounting for up to mortality rate of 48/1000 live birth in this age group [5].

The complications of malaria, including but not limited to hypoglycemia, anemia, acute kidney injury (AKI), acidosis, sepsis, hypoxia, shock, disseminated intravascular coagulopathy (DIC), respiratory distress and cerebral malaria have all been associated with poor outcomes [6]. In Tanzania most of health facilities lack the necessary resources to properly diagnose and manage acute malaria complication, some of the facilities lack basic laboratory tests to determine the acute complication of malaria [7] and this have caused the providers to rely mostly on the based on history and other clinical presentation, which may not be reliable in diagnosing the early acute complication of malaria, and hence most often patients presents or are referred late with irreversible complication of malaria [8].

The establishment of a full capacity Emergency Medicine Department at Muhimbili National Hospital (EMD-MNH) in Dar es salaam, Tanzania has provided an opportunity to improve and standardize care in the management of critically ill patients including acute complication of malaria. While most of these patients present in a critically ill stage and requires emergency interventions to abate death and improve long-term morbidity, the exact burden and outcome of patients with complications of malaria presenting to EMD remains unknown. We aimed to describe clinical presentation, resource utilization, and outcomes of acutely ill patients with complications of malaria presenting to an urban EMD in Tanzania.

## Main text

### Methods

#### Study design

This prospective study enrolled a consecutive sample of patients presenting to the EMD-MNH in Dar es salaam, Tanzania from 1 January 2017 to 31 September 2017.

#### Study setting

The EMD is a full capacity acute public emergency department of Muhimbili National Hospital (MNH), the National referral hospital located in Dar es Salaam, the commercial city of Tanzania. MNH has a 1500 bed capacity, receives an average of 1000–1200 outpatients per day, and the EMD has an annual volume of approximately 60,000 patients [9]. The department was opened in 2010, and is staffed by locally trained emergency medicine physicians who oversee care provided by medical officers, interns and residents. The department severs as the teaching unit for the only emergency medicine residency program in Tanzania [10].

#### Study protocol

The study investigator and research assistant prospectively screened and enrolled eligible patients using an electronic structured data sheet to record study information, including demographics, clinical presentation, management and EM diagnosis and final disposition. We followed up each patient to final hospital disposition, and recorded length of hospital stay, need for intensive care unit (ICU) or high dependency unit, final hospital diagnosis and clinical outcome (discharge or death).

#### Data analysis

Data from hand-written case report forms were transferred to Excel spreadsheet (Microsoft Corporation, Redmond, WA, USA), and imported to IBM SPSS Statistics v 23 for analysis. Demographic data is summarized by means and standard deviation (SD), we used Chi-square test to compare categorical variables, and Mann–Whitney U-test for comparison of continuous variables. In both comparisons, a two-tailed p-values of < 0.05 was considered statistically significant.

### Results

We screened 405 (100%) patients who were clinically diagnosed to have malaria at EMD-MNH by the treating physician. Among 405 patients, 132 (32.5%) tested positive for malaria (using either rapid diagnostic test or blood slide for malaria). In those who tested negative, 52 (19.4%) presented with at least one feature of severe malaria as defined by the WHO and National guidelines (Additional file 1: Figure S1).

#### Patient demographics

Among 405 patients screened, 184 (45.4%) were followed up (this includes 132 who tested positive for malaria, and 52 who tested negative for malaria but had at least features of severe malaria hence meeting the indication for initial malaria treatment). Among those followed up, 105 (57.1%) were male, and the overall median age was 22 years [interquartile range (IQR) of 22–32 years]. Overall 124 (67.4%) were self-referral from home, and the ITNs use among these patients was 125 (67.4%) (Table 1).

**Table 1:** Patient demographics

Demographics	Number N = 184	Percentage
	n	%
Sex		
Female	79	42.9
Male	105	57.1
Age group		
1 month to < 1 year	13	7.1
1 year to < 5 years	29	15.8
5 years to < 18 years	24	13.0
18 years to < 60 years	111	60.3
≥ 60 years	7	3.8
ITN use	125	67.9
Referral status		
Self referral	124	67.4
Referred	59	32.1
Missing	1	0.5

Clinical characteristics	n (%)	95% CI
Vital signs		
Tachycardia^b^	83 (45.1)	38.1–52.3
Febrile (T > 37.5 °C)^a^	71 (38.6)	31.9–45.8
Tachypnea^b^	56 (30.4)	24.2–37.4
Bradycardia^b^	3 (1.6)	5.6–4.7
Altered mental status	6 (3.2)	1.5–6.9
SpO_2_ < 91%	8 (4.3)	2.2–8.4
Presenting complaints		
Fever	125 (67.9)	60.9–74.3
Headache	56 (30.4)	24.2–37.4
General body malaise	41 (22.3)	16.9–28.8
Cough	15 (8.2)	5.0–13.0
Vomiting	44 (23.9)	18.3–30.6
Abdominal pain	13 (7.1)	4.2–11.7
Diarrhoea	14 (7.6)	4.6–12.4
Convulsion	26 (14.1)	9.8–19.9
Black urine	2 (1.1)	0.3–3.9

^a^All measurements in axillary

^b^Appropriate for age

#### Patients’ baseline variables and presenting complaints

Tachycardia 83 (45.1%) was the most frequent abnormal vital sign among enrolled patients; hypoxia 8 (4.3%) was least. Fever 125 (67.9%), headache 56 (30.4%) and general body malaise 41 (22.3%) were the top three presenting complaints (Table 1).

#### Investigations ordered at EMD

Of the 184 patients tested for malaria 132 (32.5) tested positive [by either rapid diagnostic malaria test (MRDT), blood slide for malaria parasite or both]. Among patients who received complete blood cell counts order 25 (13.5%) had elevated white blood counts and 21 (11.4%) had severe anaemia (Hb < 7 g/dL) of who 5 (2.7%) received blood transfusion in EMD (Additional file 2: Table S1).

#### Frequency and type of malaria complications

Anaemia 21 (11.4%) was the most frequent complication, followed by renal failure 15 (8.2%) while disseminated intravascular coagulopathy (DIC) was the least frequent complication (Table 2).

**Table 2:** Patient’s rate of complication

Complications	Overall (N = 184)	95% CI
	n/N (%)	%
Severe anaemia (Hb < 7 g/dL)	21(11.4)	7.6–16.8
Acute renal failure^a^	15 (8.2)	5.0–13.0
Respiratory distress^b^	8 (4.3)	2.2–8.3
Hypoglycemia (< 3 mmol/L)	6 (3.2)	1.5–6.9
Cerebral malaria	6 (3.2)	1.5–6.9
Shock	3 (1.6)	0.6–4.7
DIC	1 (0.5)	0.1–3.0

*DIC* disseminated intravascular coagulopathy

^a^RIFLE criteria

^b^SPO_2_ < 91% and difficulty in breathing complain

#### Patients’ disposition and hospital outcomes

Of the 184 malaria patients seen in the EMD, 99 (53.8%) were hospitalized for inpatient care and 3 patients (1.6%) died in the EMD. The overall in-hospital mortality rate was 3.8% (Table 3). The median length of stay in hospital was 1 day (IQR 1–2) days.

**Table 3:** Patients’ EMD outcome

Outcome	N = 184	95% CI
	n (%)	%
Discharged from EMD	77 (41.8)	35.0–49.1
Admitted	99 (53.8)	49.6–60.9
Died in EMD	4 (2.2)	0.9–5.5
Overall hospital mortality^a^	7 (3.8)	1.9–7.6

^a^Including EMD mortality

### Discussion

The establishment of a full capacity EMD at MNH in Tanzania manage provided an opportunity of early stabilization of acutely ill and injured patients, including those with acute complication of malaria. In this study over half of patients who were clinically suspected to have malaria by providers had neither laboratory evidence of malaria no features of severe malaria, a similar finding from what has been observed from other studies [11]. While we did not pursue further on to the treatment of this group of patients, we believe that maintaining the final EMD diagnosis of malaria, in absence of laboratory evidence and features of severe malaria, potentially put these patients in possibility of receiving antimalarial, contrary to the current national guidelines for diagnosis and management of malaria [12]. The rate of positive testing among those who were clinically suspected and tested for malaria was relatively higher compared to what has observed in other similar studies in our settings [11, 13], and we believe this might be attributed to the fact that most of these patients presents to this hospital after visiting several health facilities at district and regional levels, and hence their acuity of illness of illness is higher compared to similar studies in our settings. Interestingly, we found that majority of patients in our study population were adults above the age of 18, and were self-referral from home with low compliance to utilization of insecticide treated net (ITN). The low compliance can be attributed to many factors as seen in previous studies [14], and this know to be associate with high malaria parasitaemia, and also frequent malaria in infection in most of malaria endemic regions [15]. Fever and headache accounted for over 90% of patients presenting complaint in those who tested positive for malaria. This is contrary to what has been seen in other studies that have shown viral infections to be the major cause of undifferentiated fever in naive outpatients [16]. We believe this might have been attributed to the differences in demographics of patients seen at this study (mostly adult) and the referral patterns of patients.

Nearly half of patients in this study had features of complications of malaria. Hypokalaemia requiring replacement was observed found in 20% of patients however only 1% of patient in this study received hypokalemia management at EMD or in the wards. While we didn’t pursue the reasons for lack of treatment of hypokalemia, at the time of the study the department was quipped with management guidelines for hypokalemia, which calls for provision of supplemental potassium for these patients.

Anaemia was the most frequent complication of malaria observed in this study population, with over 10% of patients presenting with severe anaemia. Despite this presentation only one quarter of these patients received blood as part of care. This finding further complement the observation made by Shari et al. in a prospective observation of anaemic children presenting to same EMD in which only 23% of children with indications received transfusion [17]. None of patients with acute renal failure seen in this group received dialysis; all patients were managed conservatively until discharge, despite this, over 80% of these patients survived to hospital discharge. Less than 10% of patients were found to have cerebral malaria, unlike what has been observed in other studies of complications of malaria [7, 18]. One-third of patients, who were eligible to receive antimalarial treatment, did not receive it while in the EMD and we could not verify whether these patients received antimalarial while in the wards. However, several factors might have contributed to this, including the fact that some of these patients might have already received initial treatment of malaria prior to referral to this EMD.

Most patients were admitted to the wards for the inpatient care, nine patients with uncomplicated malaria were also admitted to the wards, indicating the potential for other illnesses and complications that might have been associated with current malaria diagnosis. The observed mortality rate in our study low compared to other studies of malaria done in similar settings, and this is despite the relatively short duration of hospital stay compared to other settings [19].

### Conclusions

There is a substantial burden of patients with complications of malaria presenting in the largest public ED in Tanzania. We have described their clinical presentation, management, outcomes and gaps in care provision. Future studies should focus on factors affecting delivery of appropriate care in patients with complications of malaria.

## Limitations

This was a single-centre study and our results may not necessarily be generalizable to other settings, however the EMD at MNH is the largest tertiary referral hospital in Tanzania, and receives acutely ill and injured patients from all over the country.

## Additional files

**Additional file 1: Figure S1.** Study flow diagram: patient enrollment results and outcomes.

**Additional file 2: Table S1.** Investigations ordered in the emergency department.

## Data Availability

The dataset supporting the conclusion of this article is available from the authors on request.

## Abbreviations

AKI: acute kidney injury
ED: emergency department
EMD: Emergency Medicine Department
ICU: intensive care unit
ITN: insecticide treated net
MNH: Muhimbili National Hospital
MRDT: malaria rapid diagnostic test
WHO: World Health Organisation
