# Supplementary material for: Profile and outcomes of patients with acute complications of malaria presenting to an urban emergency department of a tertiary hospital in Tanzania
Source: BMC Res Notes. 2019 Jun 18;12:345. doi: 10.1186/s13104-019-4388-8 (PMC6582575; doi:10.1186/s13104-019-4388-8)
Supplement: Supplementary file 1 — Additional file 1: Figure S1. Study flow diagram: patient enrollment results and outcomes. [file 13104_2019_4388_MOESM1_ESM.doc]

1 (2.6%)

Died

37 (97.3%)

survived

1 (1.1%)

Died

2 (4%)

Died

93 (98.9%)

survived

50 (96%)

survived

38(28.7%)

Features of complications of malaria
positive

52(19.0 %)

Features of complications of malaria

94 (71.2%)

NO Features of complications of malaria

132(32.5%)

malaria test positive

405

Clinical suspicion of malaria

221 (81%) *Excluded

273(67.5%)

malaria test negative

* *Malaria test negative Plus NO features of complications of malaria*
