# Supplementary material for: Profile and outcomes of patients with acute complications of malaria presenting to an urban emergency department of a tertiary hospital in Tanzania
Source: BMC Res Notes. 2019 Jun 18;12:345. doi: 10.1186/s13104-019-4388-8 (PMC6582575; doi:10.1186/s13104-019-4388-8)
Supplement: Supplementary file 2 — Additional file 2: Table S1. Investigations ordered in the emergency department. [file 13104_2019_4388_MOESM2_ESM.docx]

**Table S1. Investigations ordered in the EMD**

| **Laboratory Tests** | **N=184** | **(95%CI)** |
| --- | --- | --- |
|  | **n (%)** | **%** |
| MRDT positive* | 126 (68.5) | 61.5-74.8 |
| Blood slide for malaria positive | 28 (15.2) | 10.8-21.1 |
| Hypokalemia (<3.5mEq/L) | 37 (20.1) | 15.0-26.5 |
| WBC ^Ω^ (> 11 K/uL or < 4 K/uL)** | 25 (13.6) | 9.4-19.3 |
| Haemoglobin (Hb< 7g/dL) | 21 (11.4) | 7.6-16.8 |
| Elevated RFT^ε^ | 21(11.4) | 7.6-16.8 |
| Low RBG^η^ (< 3mmol/L) ^η^ | 4 (2.2) | 0.9-5.5 |
|  |  |  |
| **Treatment given at EMD** | **n (%)** | **%** |
| Antimalarial | 121 (65.8) | 58.6-72.2 |
| Intravenous fluids (NS or RL) | 81 (44.0%) | 37.0-51.2 |
| Antibiotics | 74 (40.2) | 33.4-47.4 |
| Dextrose (5% or 10%) | 42 (22.8%) | 17.4-29.4 |
| Benzodiazepines^δ^ | 8 (4.3%) | 2.2-8.4 |
| Analgesic | 6 (3.3) | 1.5-6.9 |
| Oxygen | 2 (1.1%) | 0.3-3.9 |
| Intravenous Potassium | 2 (1.1%) | 0.3-3.9 |

**MRDT-Rapid diagnostic test for malaria, ** WBC-White blood count,* ^η^ *RBG-Random blood glucose* ^ε^*RFT-Renal function tests,* ^δ^ *Diazepam or midazolam*
